# Ferroptosis in diabetic retinopathy: from pathogenic mechanisms to translational prospects

**DOI:** 10.3389/fendo.2026.1765089

**Published:** 2026-02-03

**Authors:** Jieyu Jiang, Zhimin Liu, Xiangdong Chen

**Affiliations:** 1 The First Hospital of Hunan University of Chinese Medicine, Changsha, Hunan, China; 2 Hunan University of Chinese Medicine, Changsha, Hunan, China

**Keywords:** diabetic retinopathy, ferroptosis, oxidative stress, amino acid metabolism, lipid peroxidation, pathogenic mechanism, reactive oxygen species

## Abstract

Diabetic retinopathy (DR) is a common microvascular complication of diabetes. Despite ongoing revisions in the prevention and treatment of DR, optimal treatment strategies have yet to be established. Revealing the pathological changes and molecular mechanisms of DR is the cornerstone for exploring new therapeutic strategies. Ferroptosis, a new type of programmed cell death proposed in recent years, is characterized mainly by reactive oxygen species and iron-mediated lipid peroxidation. As studies progress, growing evidence has highlighted the involvement of ferroptosis, a newly identified programmed cell death pathway, in the development and pathological mechanisms of DR. The purpose of this review is to discuss the known underlying mechanisms of ferroptosis and elucidate its role in the pathogenesis of DR. Additionally, it explores the abnormal manifestations of iron metabolism and related signaling pathways in DR. Finally, we also summarize the potential compounds that may act as ferroptosis inhibitors in DR in the future. By synthesizing these aspects, this review aims to provide insights for a deeper understanding of the relationship between ferroptosis and DR, as well as potential prevention and treatment strategies.

## 1 Introduction

Diabetic retinopathy (DR), the most prevalent microvascular complication of diabetes in the ocular fundus, severely compromises patients’ visual function and quality of life. It ranks among the leading causes of irreversible vision impairment and blindness worldwide. The pathogenesis and pathological progression of DR remain incompletely elucidated but are believed to involve multifactorial mechanisms such as inflammatory damage, ischemia, hypoxia, and oxidative stress (1). The blood-retinal barrier (BRB) plays an essential role in maintaining retinal homeostasis. Breakdown of the BRB represents a fundamental pathological event in DR, which is preceded by early core pathological changes including pericyte loss, endothelial cell dysfunction, and basement membrane thickening. These alterations disrupt retinal microcirculation, leading to increased capillary endothelial permeability, extravasation of plasma proteins, and accumulation of advanced glycation end products (AGEs) in the retinal tissue. As the disease advances, retinal hypoxia-ischemia exacerbates, triggering a cascade of pathological alterations including capillary occlusion, aberrant intraretinal and subretinal neovascularization, and neuroretinal degeneration (2).

Ferroptosis is a distinct form of regulated cell death that is mechanistically and morphologically distinguishable from apoptosis, necrosis, and autophagy (3). This iron-dependent programmed cell death process is characterized by the accumulation of lethal lipid peroxides. Cells undergoing ferroptosis display unique ultrastructural features, such as shrinkage or disappearance of mitochondrial cristae, increased mitochondrial membrane density, and rupture of the plasma membrane. Key biochemical hallmarks include dysregulated iron homeostasis, massive reactive oxygen species (ROS) generation, and suppression of the cystine/glutamate antiporter system (System Xc^-^). The core regulatory axes of ferroptosis primarily involve redox balance, lipid metabolism, iron regulation, and mitochondrial function (4–6).

Accumulating evidence indicates that ferroptosis critically contributes to the progression of diabetes and its complications via mechanisms involving lipid peroxidation and inflammation-activated neovascularization (7, 8). Substantial studies have documented the occurrence of ferroptosis in clinical DR specimens, as well as in experimental DR animal and cellular models (9–12). Targeting ferroptosis may thus represent a novel therapeutic avenue for DR. In this review, we systematically examine the molecular mechanisms of ferroptosis, discuss its pathophysiological implications and associated signaling networks in DR, and evaluate the potential of ferroptosis inhibition as a treatment strategy for DR.

## 2 Molecular mechanisms of ferroptosis: core regulatory pathways

### 2.1 Regulatory association between iron metabolism imbalance and ferroptosis

Iron, an essential element for living organisms, serves as a crucial trace element in numerous biochemical reactions and plays a central role in fundamental physiological processes such as DNA synthesis, oxygen transport, and cellular energy production. Cellular iron homeostasis generally depends on the coordinated regulation of iron uptake, export, and utilization. Disruption of iron metabolism can lead to excessive intracellular iron accumulation, which promotes the generation of ROS, causing damage to cells, tissues, and organs, and potentially triggering a unique form of regulated cell death known as ferroptosis.

**Figure 1:**
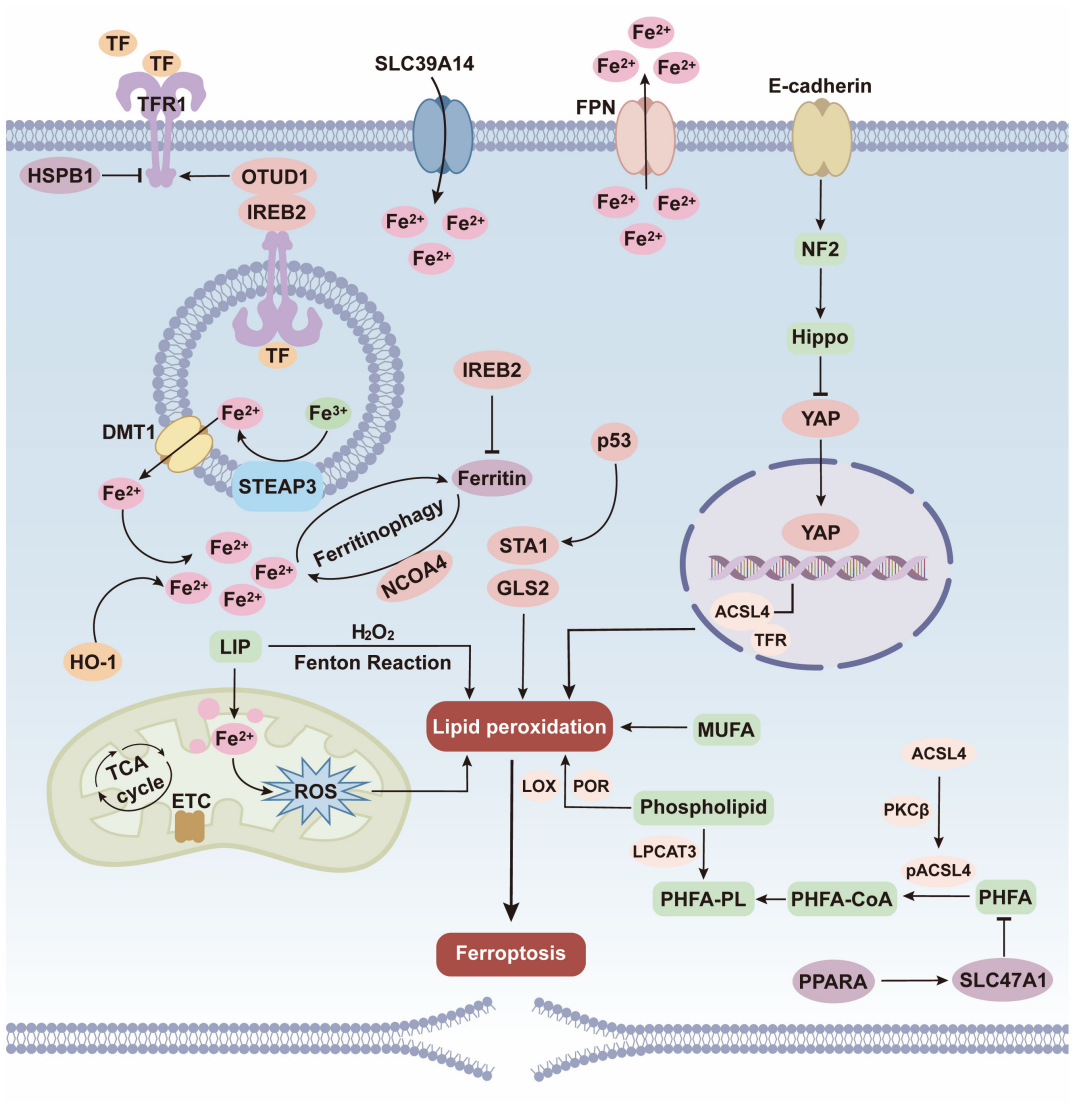
Metabolic substrate regulation mechanisms of ferroptosis. At the iron substrate level, transferrin (TF) - transferrin receptor 1 (TFR1) - mediated iron uptake and ferritinophagy - released Fe²^+^ form the labile iron pool (LIP), and Fe²^+^ generates reactive oxygen species (ROS) via the Fenton reaction. At the lipid substrate level, acyl-coA synthetase long-chain family member 4 (ACSL4) mediates the lipidation of polyunsaturated fatty acids (PUFAs), and enzymes such as lipoxygenase (LOX) promote the peroxidation of phospholipid - bound PUFAs. Meanwhile, the Hippo/YAP and p53 pathways regulate TFR and ACSL4 - related molecules. Ultimately, under the imbalance of metabolic substrate homeostasis, the accumulation of lipid peroxidation induces ferroptosis.

Physiologically, Fe³^+^-bound transferrin (TF) is internalized via transferrin receptor 1 (TFR1), reduced to Fe²^+^ and transported by divalent metal transporter 1 (DMT1) into the labile iron pool (LIP) or ferritin (13–16). LIP saturation releases free Fe²^+^ to trigger Fenton reaction, ROS production, LPO and ferroptosis; excess iron is stored or exported via ferroportin (FPN) (17–19). Encoded by SLC40A1, FPN is the only mammalian iron exporter that oxidizes Fe²^+^ to Fe³^+^, maintaining systemic iron homeostasis and regulating ferroptosis (20, 21). This sophisticated transport system effectively balances iron utilization and storage while preventing iron-mediated oxidative damage (**Figure 1**).

Iron metabolism regulates ferroptosis mainly via LIP modulation (22). Studies indicate that suppressing ferritin expression elevates intracellular LIP levels and enhances cellular susceptibility to ferroptosis. Conversely, iron chelators or ferritin and its derivatives can significantly inhibit ferroptosis by either reducing intracellular iron levels or suppressing the activity of iron-containing metalloenzymes that catalyze lipid peroxidation (23, 24). Regulation of iron uptake represents a key mechanism for maintaining intracellular iron pool capacity. Iron ions enter cells via the TF/TFR1 system or the SLC39A14 channel. Upregulation of these transporters or hyperactivation of TFRC (the gene encoding TFR1) can induce intracellular iron overload, thereby promoting ferroptosis to varying degrees (25). Notably, heat shock protein β-1 (HSPB1) inhibits TFR1, reduces iron uptake, and maintains iron pool homeostasis (26–28). Additionally, iron-responsive element-binding protein 2 (IREB2), the central post-transcriptional regulator of iron metabolism, represses ferritin synthesis, boosts TFR1 expression, and simultaneously suppresses the iron exporter FPN1. This triple action dismantles iron sequestration, promotes iron uptake, and curtails iron efflux, raising cytosolic free Fe²^+^ and supplying the critical iron pool for ferroptosis.

Furthermore, heme oxygenase-1 (HO-1) critically regulates iron metabolism (29). Sustained HO-1 expression in non-erythroid cells perturbs iron homeostasis to mediate adaptive responses, and pro/anti-inflammatory signals modulate HO-1/iron exporter expression to control local iron levels in tissues (30, 31). Elevated HO-1 correlates with reduced ferroportin and increased ZIP14/pro-hepcidin, altering ferritin expression, inducing oxidative stress and suppressing proliferation (32). Notably, the HIF-2α/IRP1 axis coordinately regulates iron metabolism genes and ferroptosis-sensitizing factors (33). HIF-2α upregulates iron/lipid metabolism genes to enhance ferroptosis sensitivity, promoting iron-dependent ROS production and cysteine oxidation-induced death. IRP1 facilitates ferroptosis by maintaining iron homeostasis and regulating TFRC, FPN, FTH1 (34).

Iron plays an essential physiological role in the visual process. It helps maintain the antioxidant balance of retinal tissue by assisting in the clearance of free radicals, thereby protecting retinal structures from oxidative stress damage. However, disordered iron metabolism can lead to abnormal iron deposition and trigger lipid peroxidation, a mechanism that may be closely associated with the pathological progression of DR (35, 36).

### 2.2 Ferroptosis mechanisms mediated by lipid metabolism disorders

Lipid peroxidation stands as one of the core mechanisms driving ferroptosis. The lipid metabolism pathway—encompassing lipid synthesis, degradation, storage, conversion, and utilization—serves as a key regulator of cellular sensitivity or tolerance to ferroptosis. Cellular and organelle membranes are enriched with polyunsaturated fatty acids (PUFAs), which act as primary substrates for lipid peroxidation (37). Notably, both the concentration and intracellular distribution of PUFAs directly influence the extent of lipid peroxidation. The chemically reactive carbon-carbon double bonds in PUFAs enable their esterification into membrane phospholipids, followed by subsequent oxidation; this cascade of reactions ultimately propagates ferroptosis signals (38).

During lipid remodeling, two core enzymes—acyl-CoA synthetase long-chain family member 4 (ACSL4) and lysophosphatidylcholine acyltransferase 3 (LPCAT3)—drive PUFA-dependent ferroptosis (39). ACSL4 expression levels directly determine the concentration of specific lipid peroxides and indirectly modulate cellular susceptibility to ferroptosis. Phosphatidylethanolamine-binding protein 1 (PEBP1) is another critical regulator of lipid peroxidation. Knockdown of ACSL4, LPCAT3, or PEBP1 suppresses PUFA synthesis and inhibits ferroptosis (40). Multiple studies have confirmed a positive correlation between ACSL4 expression and cellular sensitivity to ferroptosis (37, 41, 42). Thus, downregulating ACSL4 expression may represent a key strategy for reducing cellular susceptibility to ferroptosis. Lipoxygenases (LOXs) are widely recognized as central regulators of lipid peroxidation and ferroptosis. All LOX isoforms promote PUFA peroxidation, and their knockdown has been consistently shown to attenuate ferroptosis (43). As previously noted, PEBP1 interacts with 15-LOX to guide the oxidation of membrane-bound PUFAs, further amplifying ferroptosis. This discovery further enriches the theoretical framework underlying lipid peroxidation in ferroptosis. High glucose suppresses retinal hepcidin, causing iron overload. Free iron fuels Fenton chemistry to generate ROS that assault polyunsaturated fatty acids and ignite lipid peroxidation. LOX is up-regulated, hydroperoxide removal is blocked, and retinal cells undergo ferroptosis. Lipid dyshomeostasis produces lipid droplets that further trap iron, completing a vicious cycle.

As essential components of cell membranes, PUFAs further play a pivotal role in regulating retinal lipid metabolism and exerting antioxidant effects. Such as docosahexaenoic acid (DHA) and eicosapentaenoic acid (EPA), are indispensable for visual development and retinal function. DHA is the most abundant omega-3 fatty acid in retinal photoreceptor cells, where it is essential for maintaining structural integrity and normal physiological function. EPA, meanwhile, mitigates inflammatory responses and reduces the risk of ocular diseases such as diabetic retinopathy degeneration. Furthermore, omega-3 PUFAs protect retinal tissues by alleviating oxidative damage through their intrinsic antioxidant mechanisms (44, 45).

Beyond PUFAs that chiefly tune ferroptosis via lipid peroxidation, dysregulated metabolism of other lipid species can also directly or indirectly modulate ferroptosis. For instance, sterol lipids (including cholesterol) can undergo oxidation within cell membranes or low-density lipoprotein (LDL) particles; cholesteryl esters, a derivative of cholesterol, are also susceptible to oxidation by LOXs (46, 47). These observations suggest a potential link between cholesterol oxidation and ferroptosis. Inner BRB breakdown lets lipoproteins leak into retina, raising retinal cholesterol, while the outer BRB—retinal pigment epithelium (RPE)—transports cholesterol from choroid. Retinal cholesterol arises from local synthesis or RPE uptake of choroidal lipoproteins. Disturbed cholesterol metabolism spurs sphingolipid accumulation, down-regulates SLC7A11, curbs glutathione (GSH) synthesis and sensitizes retinal cells to ferroptosis. Thus, diabetic cholesterol dyshomeostasis—inner BRB leak plus reduced conversion to soluble oxysterols by RPE and neuroretina—elevates retinal cholesterol and drives DR (48–52). Injecting heavily oxidized/glycated LDL reproduces human DR features: vascular leakage, ERG dysfunction, vascular endothelial growth factor (VEGF) overexpression, inflammation, ER stress and apoptosis (48, 53). Cholesterol is the end product of the mevalonate pathway, and other intermediates of this pathway—particularly squalene—may also regulate ferroptosis (54). Previous studies have demonstrated that squalene acts as an effective oxygen scavenger, inhibiting the propagation of free radical reactions (55). However, it is noteworthy that squalene is typically undetectable in human cell lines, and its protective effects may only be observed under specific experimental conditions or at high concentrations (56). Dysregulation of ceramide metabolism in the diabetic retina is well documented *in vivo* and *in vitro* (57–59).Tight junctions (TJs) seal the BRB; once they break, permeability skyrockets. Lipid control of TJ integrity has been postulated for decades but remains sketchy (60, 61). Free fatty acids (FFAs) are validated diabetic threats that tilt redox balance, raise ROS and cripple endothelium (62). Palmitate (PA), the most abundant saturated FFA, triggers oxidative damage in retinal ganglion cells and fuels DR (63). PA drives Keap1 palmitoylation in human retinal microvascular endothelial cells (HRMECs); si-Keap1 reverses the ensuing Keap1/ACSL4 surge, MDA-ROS rise, Nrf2-GPX4 and GSH drop, and lessens DR and ferroptosis in mice (64). Thus, PA magnifies ferroptosis via a palmitoylation-Keap1-Nrf2/GPX4 axis, offering a new metabolic-epigenetic cross-talk route in hyperglycaemic retinal injury.

Conversely, higher plasma monounsaturated FAs (MUFAs) track with better insulin sensitivity and lower diabetes risk (65). Multi-model lipidomics first show seven MUFAs inversely relate to DR, with oleic acid (OA) the top contributor. OA curbs high-glucose-driven TLR4-MCP-1 signaling, cuts VEGF output and calms retinal inflammation (66). However, PDR patients show even higher plasma OA. This suggests that OA has dual roles depending on dose and context. Single-omics or single-model studies cannot fully reveal its nature (67).

### 2.3 Interactive regulation between amino acid metabolic pathways and ferroptosis

Amino acid metabolism supplies essential biomolecules for human physiological functions, including proteins, energy substrates, glutathione, and neurotransmitters. The amino acid-based antioxidant system primarily modulates ferroptosis via the Xc^-^ system/glutathione (GSH)/glutathione peroxidase 4 (GPX4) axis (68). Ferroptosis is triggered by the excessive accumulation of lipid peroxides, which impair membrane integrity and ultimately result in cell lysis and death. GSH and its dependent enzyme, GPX4, are core molecules that reduce lipid peroxides and safeguard cells against ferroptosis. Intracellular free cysteine levels directly dictate GSH biosynthetic capacity. Thus, cysteine modulates ferroptosis by regulating GSH production (69).

The Xc^-^ system is a heterodimeric amino acid antiporter localized on the cell membrane, consisting of two subunits—SLC7A11 and SLC3A2—linked by a disulfide bond. This system mediates the exchange of extracellular cystine for intracellular glutamate. Incoming cystine is subsequently converted to cysteine, which serves as a substrate for GSH synthesis (70). Beyond supporting GSH biosynthesis, this cysteine also activates the mammalian target of rapamycin complex 1 (mTORC1) and upregulates GPX4 protein synthesis through the Rag–mTORC1–4EBP signaling axis. Under cystine deprivation, mTORC1 activity is inhibited, leading to reduced GPX4 protein levels and thereby enhancing cellular sensitivity to ferroptosis (71).

**Figure 2:**
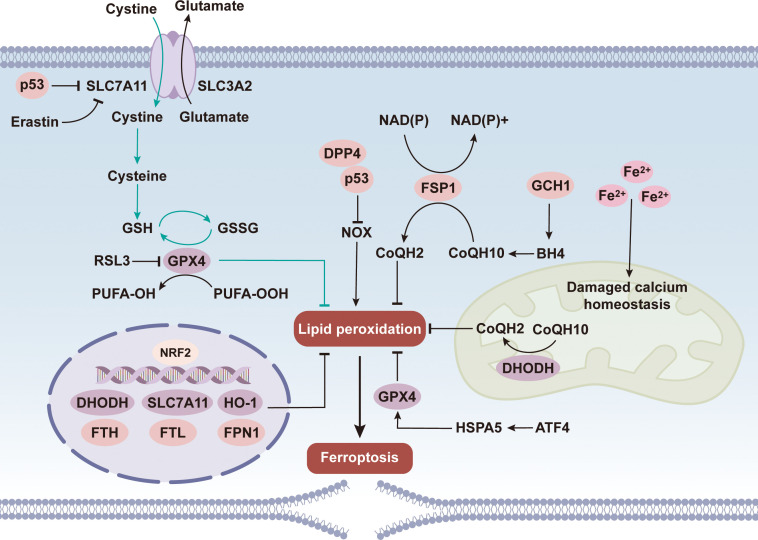
Core inhibitory mechanisms of ferroptosis. The core inhibitory mechanisms of ferroptosis center on the regulation of lipid peroxidation. System Xc^-^ mediates cystine uptake and its metabolism into glutathione (GSH), and glutathione peroxidase 4 (GPX4) relies on GSH to clear peroxides of polyunsaturated fatty acids (PUFAs). Ferroptosis suppressor protein 1 (FSP1), the gtp cyclohydrolase 1-tetrahydrobiopterin-coenzyme Q (GCH1 - BH4 – CoQ) system, and mitochondrial dihydroorotate dehydrogenase (DHODH) can also inhibit lipid peroxidation. Nuclear factor erythroid 2-related factor 2 (NRF2) enhances the inhibitory effect by transcriptionally regulating molecules such as solute carrier family 7 member (SLC7A11) and GPX4, while p53 inhibits SLC7A11 to weaken this pathway. Erastin and RSL3 target SLC7A11 and GPX4, respectively, to block the inhibitory effect, and ultimately, the accumulation of lipid peroxidation induces ferroptosis.

As a member of the glutathione peroxidase family, GPX4 is the first identified core suppressor of ferroptosis (**Figure 2**). It is the only selenoprotein capable of catalyzing the reduction of oxidized biological lipids (72). Inhibition of GPX4 activity abrogates the clearance of accumulated lipid peroxides and mitigates their cytotoxic effects, ultimately inducing ferroptosis (73). Currently, it is well established that cells counteract ferroptosis primarily through two parallel mechanisms centered on GPX4 or ferroptosis-suppressor protein 1 (FSP1). In this process, GSH acts as an essential substrate for GPX4, facilitating its reductive reactions and protecting cells from oxidative injury (74–76). Consequently, inhibition of GSH synthesis, impairment of GPX4 function, or certain pathophysiological conditions can all trigger ferroptosis. Inhibition of Xc^-^-mediated cystine uptake, which impairs GSH synthesis, is the primary mechanism of erastin-induced ferroptosis. Erastin indirectly inactivates GPX4, impairs cellular antioxidant defense, and causes cytoplasmic lipid ROS accumulation, thereby triggering ferroptosis (77). GPX4 plays a critical role in protecting retinal cells against oxidative stress and ferroptosis-associated damage (78). By catalyzing the GSH-dependent reduction of lipid peroxides, it aids in preserving cell membrane integrity against oxidative injury—an essential process for maintaining retinal homeostasis (79). In DR, downregulation of GPX4 may exacerbate oxidative stress and compromise the function and survival of retinal photoreceptor cells (80, 81).

Unlike erastin, RSL3—another classical ferroptosis inducer—does not act via GSH depletion. Instead, it binds covalently to the selenocysteine active site of GPX4, inactivating the enzyme and triggering lipid ROS accumulation (82–84). Thus, even under normal cysteine and GSH levels, direct inhibition of GPX4 can induce ferroptosis. In summary, GPX4 acts as a central regulator of ferroptosis, and the GSH antioxidant system plays a pivotal role in GPX4-mediated ferroptosis regulation. Notably, while most studies indicate that cysteine modulates ferroptosis via its role in GSH synthesis, inhibiting GSH synthesis alone is insufficient to induce ferroptosis—suggesting that cysteine may also regulate this process through alternative metabolic pathways (85). Beyond amino acids involved in GSH and GPX4 biosynthesis, additional amino acids are linked to ferroptosis. Studies have demonstrated that in experiments involving the deprivation of 14 distinct amino acids, the absence of glutamine, lysine, valine, methionine, and arginine inhibited erastin2-induced ferroptosis, whereas deprivation of glycine, tryptophan, phenylalanine, and serine had no significant impact (86–88).

Metabolomic profiling reveals that serum metabolites with significant differential expression between DR and NDR cohorts are predominantly amino acids, underscoring amino acid metabolic perturbation as a core DR-associated pathophysiological event that offers novel insights into disease pathogenesis (89). Retinal iron overload in the DR microenvironment elicits excessive production of ROS. On one hand, ROS-mediated damage to the cystine-glutamate antiporter SLC7A11—expressed on RMECs, RPE cells, and Müller cells—impairs cystine-cysteine biotransformation, disrupts the GSH- GPX4 antioxidant axis, and thereby triggers ferroptosis in these cell populations. Ferroptotic injury subsequently compromises BRB integrity, exacerbates iron accumulation and lipofuscin deposition, and culminates in retinal nerve fiber layer thinning and visual dysfunction. On the other hand, ROS downregulate taurine transporter (TAUT) expression, activate the mTOR signaling pathway in RMECs and Müller cells, and induce branched-chain amino acid (BCAA) metabolic imbalance. These perturbations synergistically attenuate cellular antioxidant capacity, promote iron retention, and amplify chronic retinal inflammation. Furthermore, iron overload upregulates arginase I in RMECs, accelerating arginine catabolism, reducing nitric oxide (NO) biosynthesis, and suppressing FPN1-mediated iron efflux to form a self-reinforcing vicious cycle (90–97). Key alterations in amino acid content and interconversion are summarized in **Table 1**.

**Table 1:** Key alterations in amino-acid levels and interconversion under diabetic retinopathy conditions.

Amino acid class	Representative amino acids	Content change	Interconversion alteration	Functional outcome in DR
Sulfur-containing	Cystine, Cysteine, Methionine	Cystine/cysteine ↓; Methionine imbalance	SLC7A11-impaired cystine→cysteine; Disrupted methionine methylation	Reduced GSH; GPX4 dysfunction; RPE ferroptosis
Taurine	Taurine	Intracellular taurine ↓	TAUT downregulation→impaired taurine uptake	Diminished iron chelation/antioxidation; RPE senescence, photoreceptor apoptosis
Branched-chain (BCAAs)	Leucine, Isoleucine, Valine	BCAA accumulation in Müller cells	Increased BCAA catabolism; Reduced BCAA→glutamine	mTOR activation; Retinal inflammation; Impaired nutrient supply
Urea cycle-related	Arginine	Arginine ↓ in RMECs	Arginase I upregulation→accelerated arginine catabolism	Reduced NO; BRB disruption; Impaired iron efflux (FPN1 ↓)

### 2.4 Coenzyme Q-dependent ferroptosis inhibitory pathways

#### 2.4.1 Molecular mechanism of FSP1-CoQ10-NADPH pathway in regulating ferroptosis

Ubiquinone (CoQ), a class of redox-active lipid molecules, plays a core regulatory role in maintaining intracellular homeostasis. FSP1 belongs to the family of glutathione-independent ferroptosis-regulating proteins (98–101). This protein can effectively counteract the ferroptotic process caused by the deficiency of GPx4 expression. Furthermore, FSP1 can mediate the regeneration cycle of CoQ10 relying on NADPH, thereby forming a functional synergy with the GPx4-glutathione antioxidant system (102). GPx4 dysfunction rapidly activates the FSP1-CoQ10-NADPH pathway.

Bersuker’s team confirmed that the core function of FSP1 is tightly linked to its myristoylation modification. This post-translational modification enables FSP1 to assemble into plasma and endoplasmic reticulum membranes, and myristoylation site mutations or aberrant modification markedly impair its ferroptosis-inhibiting activity. *In vitro* assays demonstrated that FSP1-knockout cells show heightened sensitivity to GPx4 inhibitors, further validating FSP1 as a key component of the ferroptosis defense system (103). In addition, study demonstrated that FSP1-knockout mice survive normally with no obvious phenotypes, consistent with GPx4’s compensatory lipid peroxidation inhibition, indicating functional complementarity between FSP1 and GPx4 (104). In summary, FSP1-CoQ10-NADPH forms an independent parallel defense system, complementing the classical glutathione-GPX4 pathway to inhibit phospholipid peroxidation and block ferroptosis.

In the physiological process of retinal injury repair, FSP1 also exerts a key regulatory function. This protein can provide core driving force for the repair and healing of damaged ocular tissues by upregulating the expression levels of cell adhesion molecules such as the integrin family and enhancing cell migration activity. It maintains RPE cells polarity via cytoskeletal regulation, participates in ZO-1/Claudin-1 synthesis to preserve RPE integrity and retinal function. In ocular neovascular diseases, FSP1 modulates vascular endothelial cell proliferation/differentiation/migration to regulate pathological neovascularization and scar formation (105). In addition, in the pathological progression of DR, FSP1 targets retinal inflammation and fibrous scar deposition (106). FSP1’s transcellular multi-pathway regulatory network confirms its core role in ferroptosis defense and multifaceted regulation of ferroptosis pathological sensitivity, providing theoretical support for DR pathogenesis research and therapeutic target screening.

#### 2.4.2 Mitochondrial ferroptosis defense mechanism mediated by DHODH-CoQH2 pathway

Dihydroorotate dehydrogenase (DHODH), as the key rate-limiting enzyme in the pyrimidine nucleotide synthesis pathway, not only participates in nucleic acid metabolism but also catalyzes the reduction of CoQ10 to reduced coenzyme Q10 (CoQ10H2) on the inner mitochondrial membrane, thereby exerting antioxidant function (107). Studies have shown that DHODH, GPX4 and FSP1 form three major cellular protective systems with coordinated defense via distinct subcellular localizations (108). In mitochondrial ferroptosis defense, DHODH and mitochondrial GPX4 functionally complement: DHODH blocks lipid peroxidation initiation by sustaining CoQ10H_2_ production, and GPX4 scavenges lipid peroxides to maintain mitochondrial membrane stability. Acute mitochondrial GPX4 inactivation upregulates DHODH metabolic flux, accelerating CoQ10H_2_ synthesis to neutralize lipid radicals, compensate for GPX4 deficiency and inhibit mitochondrial inner membrane ferroptosis (109). Notably, GPX4 or FSP1 cannot substitute for the protective role of DHODH, highlighting the uniqueness of the mitochondrial localized defense system. Studies demonstrated that type I interferon enhances manganese-induced ferroptosis sensitivity by downregulating mitochondrial DHODH, elucidating the underlying molecular mechanism. Consistently, the DHODH inhibitor Brequinar specifically promotes ferroptosis in GPX4-low-expressing cells by reducing mitochondrial CoQ10H_2_ levels (110). The DHODH-mediated ferroptosis defense mechanism not only deepens the understanding of the association between pyrimidine metabolism and cellular redox homeostasis but also provides experimental basis for targeting mitochondrial antioxidant systems in disease treatment, and exhibits potential value in intervening GPX4 deficiency-related diseases.

#### 2.4.3 Regulatory role of GCH1-BH4-CoQ10 pathway in ferroptosis

Guanosine triphosphate cyclohydrolase 1 (GCH1) is the rate-limiting enzyme in the synthesis of tetrahydrobiopterin (BH4) and also acts as an alternative pathway for inhibiting ferroptosis that is independent of the GPx4-GSH cysteine axis (111). Studies have shown that the GCH1/BH4 axis plays a crucial role in ferroptosis, and this role is independent of the GSH/GPX4, FSP1/CoQ10, and DHODH/CoQH2 axes (112). Photoreceptor cells are prone to oxidative damage. Relevant studies have shown that GCH1 activity in the retina is crucial for protecting photoreceptor cells from light-induced damage (113). However, the molecular targets of BH4-mediated direct antioxidant effect remain undefined, and specific research in the hyperglycemic and hypoxic pathological microenvironment of DR is lacking, with the heterogeneous role of BH4 in retinal cells yet to be further elucidated.

### 2.5 Core role of mitochondrial dysfunction in ferroptosis

In recent years, a growing body of research has revealed that mitochondria play a key regulatory role in the occurrence and development of ferroptosis (114, 115). Voltage-dependent anion channels (VDACs), core mitochondrial membrane proteins, mediate iron/fatty acid transmembrane transport, regulate cyto-mitochondrial iron homeostasis via iron metabolism-related protein interaction and participate in ROS generation (116). High VDAC-expressing RAS-mutant cells are more sensitive to erastin. VDAC2/3 knockdown inhibits erastin-induced ferroptosis, and erastin directly binds VDAC2 (117). VDAC opening mediates the entry of most metabolites into mitochondria, promoting mitochondrial oxidative phosphorylation and ROS production, and ultimately leading to mitochondrial dysfunction. Mitochondrial metabolic dysfunction reduces GSH production, impairs GPx4-mediated LPO clearance, and predisposes cells to ferroptosis (118). A study found that erastin-induced ferroptosis reduces glycolytic flux, associated with increased ATP synthesis and oxidative phosphorylation (119).

ROS generation and accumulation are key nodes in mitochondrial regulation of ferroptosis. Iron homeostasis imbalance elevates ROS levels, which degrade mitochondrial membrane-associated enzymes, impair mitochondrial membrane structure, and reduce mitochondrial membrane potential (3). Mitochondrial iron ions bind to sulfhydryl groups to generate free radicals, exacerbating oxidative stress, impairing mitochondrial membrane and enzyme function, and ultimately inducing cell death. In DR, high glucose-induced mitochondrial iron metabolic disorder and excessive ROS production synergistically activate retinal cell ferroptosis. Excess ROS impairs mitochondrial iron metabolic homeostasis, amplifies the Fenton reaction, exacerbates mitochondrial membrane damage and lipid peroxidation accumulation, thereby triggering retinal cell ferroptosis and accelerating BRB disruption as well as the pathological progression of DR. Studies have found that inducing mitochondrial oxidative stress in microglia can trigger ferroptosis, characterized by increased iron content in mitochondria, enhanced ROS production, and elevated lipid peroxidation levels (120). Mitochondria - targeted ROS scavengers can maintain the structural integrity and function of mitochondria, thereby resisting cellular ferroptosis (121).

Mitochondria and Ca²^+^ are tightly coupled to tune multiple metabolic events, and Ca²^+^ fluctuations modulate ferroptosis by reshaping mitochondrial function (122). Recent work shows that blocking mitochondrial ROS or curbing Ca²^+^ influx protects cells against Xc^-^-inhibitor-induced ferroptosis (123, 124). Ca²^+^ overload also activates calcineurin and other signaling molecules, thereby regulating VDAC channel activity and iron-metabolism gene expression to indirectly influence ferroptosis. However, whether Ca²^+^ drives DR-related ferroptosis via mitochondrial pathways remains largely unexplored and warrants deeper investigation.

### 2.6 Other key regulatory pathways of ferroptosis

#### 2.6.1 Nrf2 pathway-mediated antioxidant regulation of ferroptosis

As the core transcription factor for cellular antioxidant stress, Nrf2 regulates ferroptosis via multiple pathways (125). On one hand, Nrf2 activates downstream target genes to regulate iron metabolism-related genes, reducing intracellular free iron accumulation and Fenton reaction-induced oxidative damage (126–130). It upregulates GSH synthetic key enzymes and GPx4 expression, strengthening cellular LPO scavenging capacity and blocking ferroptosis (131, 132). On the other hand, oxidative stress signals reversely activate the Nrf2 pathway. ROS mediate Keap1 conformational change, relieving its inhibition on Nrf2 and enabling Nrf2 nuclear translocation to exert antioxidant defense (133).

In DR, high glucose inhibits Nrf2 activity by activating protein kinase C (PKC) and accumulating advanced glycation end products (AGEs), reducing retinal cell GSH/GPx4 levels, impairing iron export, inducing free iron and LPO accumulation, and activating ferroptosis in Müller, ARPE and endothelial cells, exacerbating vascular leakage (134–136). Therefore, activating retinal Nrf2 pathway to restore iron metabolism and lipid peroxidation regulation is a potential strategy for delaying DR progression. However, the core downstream target genes mediating Nrf2-regulated DR iron metabolism and lipid peroxidation lack in-depth validation, and the optimal activation strategy, long-term safety of Nrf2 pathway and its crosstalk with other ferroptosis regulatory pathways remain insufficiently studied, warranting further investigation.

#### 2.6.2 Regulatory role of p53 pathway in ferroptosis

p53 acts as a transcriptional repressor of SLC7A11, inhibiting cysteine uptake to promote ferroptosis (137–139). Studies show negative correlation between p53 activation and SLC7A11 expression in mouse embryonic fibroblasts (MEFs), confirming this regulatory relationship (140).

Furthermore, p53 induces SAT1 transcription to promote lipid peroxidation and ROS-dependent ferroptosis, and iron depletion upregulates SAT1 expression (141). Glutamine metabolism regulates ferroptosis: glutaminase 2 (GLS2) is a novel p53 target, and p53 promotes ferroptosis by activating GLS2 (142). It is worth noting that the regulation of ferroptosis by p53 is bidirectional. One study found that p53 promotes DPP4 nuclear translocation to form complexes, inhibiting NOX-mediated lipid peroxidation and ferroptosis; p53 deficiency allows DPP4 to bind NOX1, exacerbating lipid peroxidation and ferroptosis. This indicates p53 may also negatively regulate ferroptosis (143, 144). Currently, the subtype-specific regulation of retinal target cells by p53 under high glucose remains undefined, and the switch mechanism of p53 bidirectional ferroptosis regulation is unclarified, warranting further research on its specific mechanisms.

#### 2.6.3 Association mechanism between NADPH metabolic imbalance and ferroptosis

Nicotinamide adenine dinucleotide phosphate (NADPH), is closely associated with the pathogenesis and progression of various metabolic diseases, and plays a pivotal role in ferroptosis regulation by modulating antioxidant system function and iron metabolism homeostasis, with its intracellular levels directly dictating cellular susceptibility to ferroptosis (145, 146). The pentose phosphate pathway (PPP) is a major source of cytoplasmic NADPH, with glucose-6-phosphate dehydrogenase (G6PDH) and 6-phosphogluconate dehydrogenase (6PGDH) as core rate-limiting enzymes (147). Studies have found that knocking down G6PD or 6PGD significantly inhibits erastin-induced ferroptosis, suggesting PPP-derived NADPH participates in ferroptosis occurrence (148).

In fundus diseases, NADPH regulates ferroptosis to modulate pathological progression (149). DR retinal chronic inflammation amplifies RPE ferroptosis: inflammatory factors activate NOX to produce ROS via NADPH, damaging antioxidant systems and inducing ferritinophagy to release free Fe²^+^, further enhancing ferroptosis signals (150). Current research has confirmed that supplementing NADPH precursors, iron chelators, or inhibiting NOX activity effectively alleviates ferroptosis of DR. Targeting the NADPH metabolic pathway is a promising therapeutic strategy for DR. However, the optimal dosage, administration route and long-term safety of NADPH precursors remain unvalidated in large animal models, and the crosstalk of NADPH metabolism, iron homeostasis and retinal inflammation is poorly understood, limiting clinical translation of NADPH-targeted therapies and requiring further investigation.

## 3 Synergistic pathogenic mechanisms of ferroptosis in DR

### 3.1 Vicious cycle mechanism of oxidative stress-BRB injury-ferroptosis

Oxidative stress is defined as an imbalance between reactive oxygen species (ROS) and the endogenous antioxidant defense system, and the retinal tissue and cellular damage it mediates serves as a core pathological driver of diabetic retinopathy (DR) progression (151). Diabetic patients present with chronic hyperglycemia, and elevated glucose levels together with canonical hyperglycemia-associated metabolic pathways (polyol pathway, hexosamine pathway, protein kinase C activation, and advanced glycation end product accumulation) induce excessive ROS production to trigger oxidative stress (152–155). Excessive ROS not only directly causes oxidative damage to retinal cells and initiates retinal neovascularization, but also impairs the structural and functional integrity of the BRB. BRB disruption is a primary pathogenic mechanism of DR and the key factor driving DR progression from non-proliferative DR (NPDR) to proliferative DR (PDR). Under physiological conditions, the BRB coordinates vascular and extravascular systems to maintain osmotic balance, regulate ion concentrations, and facilitate nutrient/metabolite transport, thereby preserving retinal microenvironmental homeostasis. Retinal ischemia-hypoxia induced by BRB impairment further triggers neovascularization and nonperfusion, which exacerbates oxidative stress dysregulation and iron metabolism imbalance, forming a vicious positive feedback loop between oxidative stress and BRB damage that accelerates DR pathogenesis and amplifies ferroptosis susceptibility via iron overload and lipid peroxidation.

Cells harbor an endogenous antioxidant system comprising superoxide dismutase (SOD) and catalase: SOD converts superoxide anions to H_2_O_2_, and catalase further reduces H_2_O_2_ to water. In diabetes, retinal antioxidant defense enzyme activity is impaired, leading to excessive free radical accumulation (156). Hyperglycemia acts as both an initiator of oxidative stress and a co-promoter of pathogenic molecular pathways, and cross-talk among these pathways induces ROS amplification via positive feedback, exacerbating oxidative damage, BRB impairment, iron dyshomeostasis and DR progression (157, 158). Pharmacological alleviation of oxidative stress effectively ameliorates DR pathology, validating oxidative damage clearance as a promising therapeutic strategy for DR.

Ferroptosis, an oxidative stress-dependent regulated cell death modality, is potently triggered by oxidative stress, a mechanism particularly critical in ocular pathologies (159).Accumulating evidence implicates ferroptosis in retinal neurodegeneration and DR pathogenesis, supporting oxidative stress as a central bridge linking ferroptosis to DR progression (160, 161). Notably, ROS scavenging by N-acetylcysteine (NAC) inhibits high-glucose-induced ferroptosis, confirming that oxidative stress-mediated ferroptosis contributes to DR pathogenesis (162). A novel miR-338-3p/SLC1A5 axis has been identified as a key regulator of oxidative stress-induced ferroptosis in DR: high glucose significantly upregulates miR-338-3p expression in RPE cells, and miR-338-3p silencing reverses RPE cell death and ferroptosis (9). Mechanistically, hyperglycemia-induced miR-338-3p upregulation represses SLC1A5 expression, thereby impairing antioxidant capacity and triggering oxidative stress-mediated ferroptosis, which in turn exacerbates BRB damage and oxidative stress to form a oxidative stress-BRB impairment-ferroptosis positive feedback loop that drives DR progression. Additionally, as aROS-derived lipid, 4-hydroxynonenal (4-HNE) serves as one of the core mediators driving the pathogenesis and progression of retinal pathology induced by systemic metabolic diseases. Studies have demonstrated that the levels of 4-HNE, p53 and phosphorylated p53 are significantly elevated, whereas the levels of GSH, SLC7A11 and GPX4 are decreased in both C57BL/6J mice and cultured HRECs (11).

Although studies have confirmed that the three components form a positive-feedback loop that drives DR progression and that antioxidant therapy can alleviate DR pathology, the key molecular triggers that initiate the loop—such as the cell-specific mechanisms by which hyperglycemia induces excessive ROS production in different retinal cells—remain poorly defined. Moreover, regulatory axes such as miR-338-3p/SLC1A5 have been examined almost exclusively in retinal pigment epithelial cells; whether they operate similarly in retinal endothelial cells, pericytes, or other retinal subpopulations has not been tested. Systematic data describing how this feedback loop evolves across DR stages (non-proliferative versus proliferative) are also lacking, making it difficult to align potential interventions with the precise windows of the clinical disease course.

### 3.2 Synergistic pathogenic mechanism of inflammation-neovascularization-ferroptosis

Iron overload acts as a pivotal hub linking ferroptosis, inflammatory responses, and pathological neovascularization in DR. These three pathological processes drive the progression of DR from the non-proliferative to the proliferative stage and induce retinal neurodegeneration through cascade amplification and positive feedback regulation. In a hyperglycemic microenvironment, iron overload promotes VEGF secretion through two synergistic pathways. First, it enhances succinate accumulation and upregulates its receptor GPR91, and the activated succinate/GPR91 axis triggers VEGF production via the ERK1/2, p38 MAPK, and JNK signaling pathways. Second, it induces oxidative stress, which further amplifies VEGF overexpression (163, 164). Excessive VEGF accelerates the degradation of tight junction proteins (ZO-1, claudin-5, occludin), increases the abundance of caveolae in the membranes of RECs, and directly disrupts the BRB, subsequently inducing retinal edema. Tripartite motif-containing 46 (TRIM46) exerts dual regulatory effects on RECs dysfunction and ferroptosis initiation. By promoting the ubiquitination and degradation of GPX4, and IκBα, TRIM46 not only triggers ferroptosis via catalyzing membrane lipid peroxidation but also activates inflammatory cytokine cascades. This process not only reduces transendothelial electrical resistance (TEER) and increases RECs permeability but also further downregulates the expression of ZO-1 and claudins through the nuclear factor-κB (NF-κB) pathway, resulting in a synergistic effect on BRB disruption (165).

In addition, oxidative stress and inflammation induced by iron overload impair the signal crosstalk between pericytes and RECs, leading to pericyte loss and the complete collapse of the BRB structure (166). In RPE cells, iron overload downregulates the expression of circSPECC1 and Rpe65, which disrupts RPE cells polarity, induces ultrastructural abnormalities and atrophy, and activates microglia. These pathological alterations ultimately damage the outer BRB and accelerate RPE cell death (167). Notably, overexpression of ubiquitin-specific protease 48 (USP48) can inhibit the above pathological processes by deubiquitinating SLC1A5, suggesting that targeting the ferroptosis-inflammation axis represents a promising potential strategy for DR treatment (168). However, significant gaps persist. First, no systematic profiling of key inflammatory cytokines has been performed, so the full regulatory map of the ferroptosis–inflammation–neovascularization axis remains sketchy: the exact spectrum of cytokines released upon iron overload and their divergent effects on ferroptosis versus neovascular signaling are still undefined. Second, the molecular details of how iron overload disrupts communication between pericytes and retinal endothelial cells are poorly understood, and therapeutic strategies aimed at this cooperative vulnerability remain confined to cellular and animal models, with no clinical data on efficacy or safety.

### 3.3 Bidirectional regulatory relationship between autophagy and ferroptosis

Autophagy is a lysosome dependent physiological process that maintains homeostasis by degrading damaged macromolecules or organelles within the cell. Although ferroptosis is a novel form of programmed cell death distinct from autophagy, the two are not isolated but rather form a bidirectional regulatory relationship through multiple molecular mechanisms. Both the initiation and progression of ferroptosis involve autophagy. Autophagy can degrade iron related proteins and antioxidant molecules to promote ferroptosis, but it can also clear peroxidized lipids and damaged organelles to inhibit ferroptosis. The specific regulatory direction depends on the cell type, the intensity of stress signals, and the microenvironment (169, 170).

In a high - glucose environment, autophagy can degrade toxic ferritin through ferritinophagy to release Fe²^+^, which produces ROS via the Fenton reaction, thereby amplifying ferroptosis driven by LPO (171, 172). On the other hand, it can also clear peroxidized lipids and damaged organelles, maintain the GSH/GPx4 system, and inhibit ferroptosis (173). Studies have found that in the retinal tissue of DR patients, high glucose can induce upregulation of NCOA4 expression, enhance ferritinophagy, reduce FtL, increase LC3-II/I, and activate NOX to produce ROS, forming a “ROS – ferritinophagy-free iron” positive feedback loop (172). BECN1 and ATG7 have been identified as potential biomarkers (174).Long-term high glucose can deplete autophagy-related proteins or inhibit flux through AGEs, weakening antioxidant protection and thereby exacerbating ferroptosis. Mitophagy can both clear damaged mitochondria to reduce ROS and promote ferroptosis by inhibiting complex I or upregulating HO-1 (175).

As previously mentioned, AMPK/mTOR regulates ferroptosis in DR (176). However, whether mTOR mediates ferroptosis and regulates Müller cells in DR remains unclear. Moreover, the regulation of both processes relies on common signaling pathways, further strengthening their association in DR. For example, the nuclear transcription factor Nrf2 is both a positive regulator of autophagy and a key inhibitor of ferroptosis (177). Current studies confirm that the two processes are linked via shared pathways such as ferritinophagy, mitophagy, and Nrf2, yet many questions remain. First, the net direction of autophagy’s effect on ferroptosis—pro-death or pro-survival—depends on cell type and stress intensity, but the dominant mode in each retinal cell population within the DR micro-environment is still unclear. Second, whether mTOR itself drives ferroptosis and governs Müller-cell function in DR has not been settled, and the molecular details of how the AMPK/mTOR axis steers their crosstalk are fragmentary. Finally, most reports examine only a single autophagy subtype or pathway; integrated analyses of how different autophagic routes cooperate or compete to tune ferroptosis are missing, and therapeutic strategies aimed at their common nodes still require rigorous pre-clinical validation of specificity and efficacy.

## 4 Ferroptosis in retinal cells

### 4.1 Regulatory mechanisms and pathological roles of ferroptosis in RPE cells

**Figure 3:**
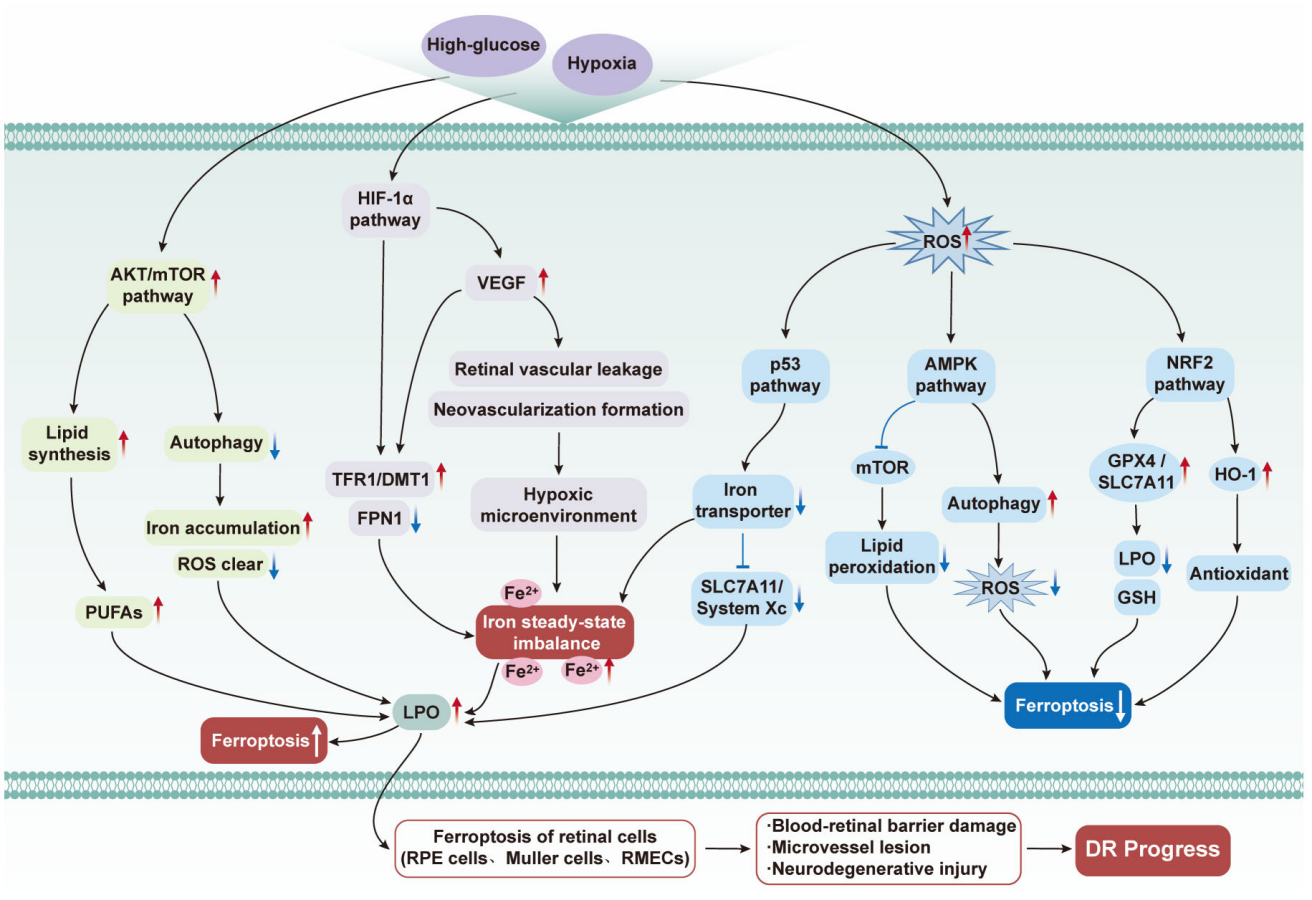
Potential mechanisms of ferroptosis in diabetic retinopathy. Hyperglycemia and hypoxia, as core microenvironmental stimuli in diabetic retinopathy (DR), trigger ferroptosis in retinal cells through multiple pathways: activating the hypoxia-inducible factor-1α (HIF - 1α) pathway to upregulate vascular endothelial growth factor (VEGF), which induces vascular leakage/neovascularization, while also activating the protein kinase B/mammalian target of rapamycin (AKT/mTOR) pathway to promote Polyunsaturated fatty acids (PUFA) accumulation and inhibit autophagy to weaken reactive oxygen species (ROS) clearance; hypoxia also disrupts iron homeostasis by upregulating transferrin receptor 1 (TFR1)/divalent metal transporter 1 (DMT1) and downregulating ferroportin 1 (FPN1), and inhibits System Xc^-^ (SLC7A11) to reduce glutathione (GSH) synthesis; excessive Fe²^+^ synergizes with PUFAs to induce lipid peroxidation (LPO), ultimately leading to ferroptosis. ROS bidirectionally regulates ferroptosis: the p53 pathway exacerbates it, while the adenosine monophosphate-activated protein kinase (AMPK)/nuclear factor erythroid 2-related factor 2 (NRF2) pathway inhibits it, and ferroptosis in retinal cells directly drives the progression of DR by disrupting the blood - retinal barrier, microvasculature, and causing neuronal damage.

Retinal pigment epithelial (RPE) cells are located between the choroid and photoreceptors, undertaking core functions such as phagocytosis of photoreceptor outer segments, transport of nutrients, and inhibition of oxidative stress. They are key cells in maintaining retinal homeostasis, and their functional impairment or death is closely related to the progression of DR and other retinal diseases (178). An increasing number of studies have observed ferroptosis in both *in vivo* and *in vitro* models of DR (179, 180). The key mechanisms of ferroptosis are closely related to the pathological mechanisms of DR, including iron homeostasis imbalance, lipid peroxidation, glutathione depletion, GPX4 inactivation, and LOX upregulation (**Figure 3**). Under normal conditions, the intracellular antioxidant system can clear excess ROS. However, in patients with DR, the activity of the antioxidant system is reduced, leading to a surge in ROS production. This is both the main cause of the phenomenon triggered by hyperglycemia and the core inducer of the decline in RPE cell vitality and function (168, 181, 182). High glucose activates the PKC pathway and promotes the accumulation of advanced glycation end products (AGEs), which on the one hand accelerates the consumption of NADPH in RPE cells. The depletion of NADPH directly leads to a decrease in GSH levels, inactivates GPx4, and prevents the clearance of intracellular LPO, eventually causing the accumulation of LPO. On the other hand, AGEs bind to the RAGE on the RPE cell membrane, disrupting iron metabolic homeostasis and accelerating the ferroptosis process in RPE cells (183–185). It has been found that treating RPE cells with high-concentration glucose solution (30 mM) increases ROS levels, promotes cell ferroptosis, and inhibits cell proliferation and vitality in RPE. However, co-treatment of cells with the ferroptosis inhibitor Ferrostatin-1 and NAC can reverse ferroptosis (9). Tang et al. found that high glucose promotes the death of ARPE-19 RPE cells, increases the levels of ROS and oxidized glutathione (GSSG), and enhances the lipid peroxidation density of the mitochondrial membrane. Adding Fer-1 to RPE cells reduces cell mortality, indicating that HG-induced oxidative damage occurs due to ferroptosis (186). Ferroptosis drives the further occurrence and development of DR through lethal ROS accumulation, iron overload, and uncontrolled lipid peroxidation, leading to RPE cell damage and death. In addition, it has been found that the miR-338-3p/SLC1A5 axis plays a key regulatory role in this process. Both knockout of miR-338-3p and overexpression of SLC1A5 can eliminate ferroptosis caused by high glucose in RPE cells (9).

Nrf2, a key transcriptional regulator involved in redox homeostasis, plays a crucial role in antioxidant stress (187). Sirt1, a class III protein deacetylase, can promote the deacetylation of histone lysine residues and regulate the activity of various transcription factors such as PGC-1α, NF-κB, and P53 (188, 189). It has been found that a high-glucose environment reduces the expression levels of Sirt1 and Nrf2 in the nuclei of retinal pigment epithelial cells and inhibits the expression of downstream Nrf2 genes such as GPX4, GCLC, and GCLM (190).

Cofilin-2 (CFL2), as a member of the actin-binding protein family, is crucial for maintaining normal muscle function (191, 192). In recent years, more functions of CFL2 have been gradually revealed. In DR, CFL2 has been proven to be overexpressed in RPE cells induced by high glucose, and its upregulation exacerbates high glucose-induced cell ferroptosis (193). Specific protein 1 (SP1) is a sequence-specific DNA-binding protein that has been proven to regulate tumor proliferation, angiogenesis, and metastasis (194). This protein can bind to the promoter regions of multiple genes associated with the progression of diabetic retinopathy, such as ROBO1 and MALAT1 (195, 196). However, whether SP1 regulates CFL2 to mediate high glucose-induced RPE cell damage remains unknown. Although existing literature has clarified the core role of RPE cell ferroptosis in DR progression and preliminarily elucidated the regulatory mechanisms of iron homeostasis imbalance, lipid peroxidation and related molecular axes, multiple limitations remain. Most studies lack sufficient detection of ferroptosis-specific phenotypes, and *in vivo* research is deficient in the localization of RPE-specific ferroptosis, dynamic changes across distinct DR stages, and validation of targeted interventions.

### 4.2 Role and mechanism of müller cell ferroptosis in DR

During the progression of DR, Müller cells play a pivotal role. Studies have found that in the early stages of diabetic retinopathy, pathological changes occur in Müller cells prior to those in retinal vascular endothelial cells and pericytes (197). Early retinal pathological changes include the activation of retinal glial cells, with the most significant being the activation of Müller cells. Activated Müller cells can release a large number of inflammatory factors and cytokines, which disrupt the stable retinal microenvironment through various molecular pathways (198). An increasing number of viewpoints now suggest that diabetic retinopathy is not merely a vascular complication but rather a neurovascular disorder. Neuroinflammation and retinal neurodegeneration are early driving factors of DR (199, 200).

Müller cells, the main glial cells of the retina, are radially distributed throughout the entire retinal layer and are considered the structural and functional link between retinal neurons and the vitreous cavity, subretinal space, and blood. They are crucial for retinal homeostasis and highly sensitive to hyperglycemia (201). Under hyperglycemic conditions, Müller cells experience oxidative stress, inflammation, and mitochondrial dysfunction, leading to neuronal damage and neuroinflammation (202–205). Recent studies have shown that hyperglycemia disrupts the iron homeostasis of Müller cells, making them susceptible to ferroptosis, characterized by lipid peroxidation, iron overload, and impaired antioxidant defenses (206, 207). Studies have shown that glucose concentrations above 25 mM trigger ferroptosis, marked by increased levels of malondialdehyde (MDA), 4-hydroxynonenal (4-HNE), and Fe²^+^, as well as altered expression of ACSL4, ALOX15, GPX4, and SLC7A11 (208, 209). Ferroptosis inhibitors can suppress lipid peroxidation, restore GPX4 expression, and enhance cell viability, while inducers exacerbate cell death (210, 211). These findings indicate that hyperglycemia induces Müller cell ferroptosis via iron overload, lipid peroxidation and Xc^-^–GSH–GPX4 axis dysfunction, which further exacerbates neuroinflammation through microglial reactivation and impairs the BRB. Notably, these studies are still mostly based on cellular or animal models with unvalidated clinical translatability, and the cross-validation of ferroptosis regulatory mechanisms remains insufficient. AQP4 positively regulates TRPV4 expression. Overexpression of TRPV4 enhances ferroptosis and oxidative stress in Müller cells treated with high glucose, while inhibition of TRPV4 has a protective effect on retinal damage caused by DR in rats (212). TRPV4 may be a potential therapeutic target for DR.

### 4.3 Ferroptosis in RMECs and vascular function injury mechanisms

The retina is in a metabolically active state due to elevated oxygen consumption, high glucose utilization, and abundant levels of PUFAs. These biochemical characteristics make ocular neural tissues more susceptible to oxidative damage compared to the whole-body biological system. RMECs, as the basic structural units of the retinal microvasculature, are an important component of the inner BRB. Since capillary endothelial cell dysfunction and death are the initiating factors of diabetic retinopathy, timely and precise management of endothelial abnormalities may delay the progression of DR (213–217). Studies have shown that endothelial dysfunction caused by oxidative stress-mediated endothelial inflammation, lipid peroxidation, and activation of PCD is the root cause of these changes (218, 219). High concentrations of VEGF can induce structural and functional changes in human retinal microvascular endothelial cells (HRMECs), leading to pathological vascular remodeling, which in turn exacerbates vascular leakage and abnormal angiogenesis (220). Studies have found that berberine can effectively intervene in the course of DR by inhibiting ferroptosis in HRMECs, and its mechanism of action may be realized through the Nrf2/HO-1/GPX4 signaling pathway (221). Similarly, studies have shown that amygdalin treatment through the NRF2/ARE signaling pathway can significantly inhibit ferroptosis and oxidative stress in HRECs stimulated by high glucose (222). A study analyzing the association between ferroptosis and DR in vitreous samples from patients with PDR confirmed that high glucose-induced ferroptosis shows a time-dependent feature in HRMECs. The study found that high glucose-induced loss of PCBP1 activates the HIF-1α/HO-1 pathway, leading to intracellular iron homeostasis imbalance and increased ferrous ion levels, while also exacerbating lipid peroxidation and ferroptosis. Moreover, overexpression of PCBP1 or direct targeting of HO-1 can effectively alleviate iron overload, lipid peroxidation, and ferroptosis in HRMECs and improve cell viability by downregulating HO-1 expression (223). These findings suggest that enhancing PCBP1 activity may become a potential new direction for the treatment of DR. Other studies have shown that TRIM46 enhances lipid peroxidation and ferroptosis in HRCECs stimulated by high glucose through promoting GPX4 ubiquitination (178). Interestingly, a study found that reduced vitamin D plays a key role in the oxidative stress and vascular endothelial injury caused by diabetes. 25 (OH) D3 may inhibit ferroptosis in HRMVECs induced by high glucose by downregulating miR – 93 (224). However, compared with traditional markers such as inflammation and apoptosis, the emerging programmed-cell-death modality of ferroptosis has received little attention in diabetic endothelial dysfunction. Work to date concentrates on canonical pathways, leaving the core ferroptotic circuits, molecular targets and regulatory networks that drive retinal microvascular endothelial-cell (RMEC) injury undefined. Moreover, associations with routine clinical endocrine indices (e.g., HbA1c) are lacking, and the efficacy and translational value of ferroptosis-targeted interventions remain to be proven—critical gaps that now demand urgent attention.

## 5 Potential therapeutic strategies targeting ferroptosis

Research on the mechanisms of ferroptosis has opened up new avenues for the treatment of DR. (**Table 2**).

**Table 2:** Mechanisms of ferroptosis - related targets in DR.

Ferroptosis-related targets	Study subject	Mechanism	Reference
Ferrous ion, ROS	ARPE-19 cells	Overexpression of USP48 deubiquitinates SLC1A, thereby promoting cell proliferation and inhibiting ferroptosis and oxidative stress.	(168)
GPX4, FTH1, Ferrous ion	ARPE-19 cells, Müller cells, C57BL/6J mice	Sestrin2 inhibits ferroptosis by suppressing STAT3 phosphorylation and endoplasmic reticulum stress.	(185, 203)
Nrf2, GPX4	ARPE-19 cells	Maresin - 1 inhibits high - glucose - induced ferroptosis in ARPE - 19 cells by activating the Nrf2/HO - 1/GPX4 pathway.	(137)
Nrf2, GPX4	ARPE-19 cells	Astragaloside IV mitigates high-glucose-induced ferroptosis in ARPE-19 cells by activating the Nrf2/GPX4 pathway; miR-138-5p exerts its effects by downregulating Sirt1/Nrf2.	(186)
GSH, Ferrous ion	ARPE-19 cells	Circular RNA PSEN1 downregulation alleviates ferroptosis in high-glucose - treated RPE cells through the miR - 306b - 5p/Atg7 axis.	(193)
SLC1A5	ARPE-19 cells	miR-338-3p targeted the 3’ untranslated regions (3’UTR) of SLC1A5 for its inhibition and degradation, and high glucose downregulated SLC1A5 by upregulating miR-338-3p in RPE cells.	(9)
4-HNE, p53, GSH, SLC7A11	C57BL/6J mice, HRECs	Overexpression of miR-214-3p leads to the downregulation of p53 as well as the upregulation of SLC7A11 and GPX4, thereby alleviating the damage induced by ferroptosis.	(11)
GPX4	ARPE-19 cells	Ferrostatin - 1 alleviates tissue and cell damage in DR by improving the antioxidant system.	(79)
Ferritin	ARPE-19 cells	BECN1, HERC2, ATG7, and BCAT2 are involved in ferritin degradation, affecting ferroptosis and proliferation.	(174)
GPX4, FSP-1	ARPE-19 cells	1,8-Cineole alleviates DR by inhibiting RPE ferroptosis via the PPAR-γ/TXNIP pathway.	(225)
Nrf2, HO-1, ROS, GSH	ARPE-19 cells	PLD can downregulate the ferroptosis - related TLR4/MyD88/NF - κB p65 pathway and upregulate the Nrf2/HO1 pathway.	(226)
GPX4, FTH1, ACSL4, TFRC	HRMECs	Under high - glucose conditions, ferroptosis is associated with elevated levels of ROS, lipid peroxides, and iron.	(220)
ACSL4, TFR1, SLC7A11, GPX4, Ferrous ion	HRMECs	Overexpression of PIM1 inhibits inflammatory responses, cell migration, and tube formation; it increases tight junction protein levels in HRMECs.	(216)
SLC7A11, GPX4	HRMECs	Ferroptosis - related genes are significantly enriched in the processes of ROS metabolism and iron ion reactions.	(160)
Nrf2, HO-1, GPX4, FSP1, ACSL4	HRMECs	Berberine can upregulate the expression of Nrf2, HO - 1, GPX4, and FSP1 while inhibiting ACSL4.	(221)
Nrf2, HO-1, GSH, GPX4, SOD, Fe2	HRECs	Amygdalin treatment alleviated high - glucose - induced damage in HRECs and promoted the nuclear translocation of NRF2 in high - glucose - stimulated HRECs.	(222)
p53, ROS, GSH, Fe2+	C57BL/J mice, HRCECs	Isoquercetin alleviated ferroptosis induced by HG in HRCECs by modulating the p53 pathway.	(227)
GPX4, SLC7A11, Ferrous ion	HRMVECs	25Hydroxyvitamin D3 alleviates high - glucose - induced oxidative stress and ferroptosis in retinal microvascular endothelial cells by downregulating GPX4.	(224)
GPX4	HRCECs	TRIM46 exacerbates high - glucose - induced retinal inflammatory responses by promoting endothelial cell injury.	(178)
GPX4-YAP	DR patients, Male C57BL/6 mice,	Pipecolic acid may hinder the progression of DR by inhibiting the YAP-GPX4 signaling pathway	(83)
GPX4, GSH, Ferrous ion	DR patients	Compared with the normal group, patients with DR have significantly lower concentrations of GPX4 and GSH, and significantly higher concentrations of LPO, Fe, and ROS.	(35)
ROS	Male SD rats	Inhibition of AQP4 can suppress ferroptosis and oxidative stress in Müller cells by downregulating TRPV4.	(212)
ACSL4	Male SD rats	Glia maturation factor - β induces ferroptosis by impairing chaperone-mediated autophagic degradation of ACSL4 in early DR.	(179)
Nrf2, HO-1, SLC7A11, SOD	Male C57BL6 mice	Resveratrol may inhibit the apoptosis of retinal ganglion cells by inhibiting the Nrf2/HO-1 pathway and reducing retinal oxidative stress.	(228, 229)
GPX4, GSH, ACSL4, Ferrous ion	db/db mice	DFO significantly reduced inflammatory marker levels in diabetic mice models and improved retinal electrophysiological function.	(230)
TFR1, SLC7A11, FSP1, VDAC	RGCs	Fer - 1 significantly reduced retinal inflammation and maintained the integrity of BRB.	(231)

### 5.1 Therapeutic potential of ferroptosis inhibitors in DR

Fer-1 is a well-known specific small molecule ferroptosis inhibitor that can eliminate lipid peroxides. This drug has shown significant protective effects in various preclinical models, including retinal cells (232). These lipophilic antioxidants can eliminate lipid peroxide radicals and prevent the spread of lipid peroxidation reactions, thereby maintaining cell membrane integrity. In the streptozotocin (STZ)-induced diabetic rat model, intravitreal injection of the ferroptosis inhibitor Fer-1 significantly reduced retinal inflammation, maintained the integrity of BRB, and prevented RGCs loss, which was confirmed 4–8 weeks after diabetes induction (232, 233). Under high glucose conditions, in 661W cells and photoreceptor cells of diabetic mice, the expression of GPX4 and SLC7A11 is downregulated, while the expression of ACSL4, FTH1, and NCOA4 is upregulated. Fer - 1 can alleviate ferroptosis and protect the retina from light - induced retinal degeneration (232). In addition, it was found that Fer-1 can reduce cell damage and the accumulation of ferrous iron in HRECs treated with high glucose, and also improve mitochondrial membrane potential and restore GSH levels (179, 230). However, unfortunately, although *in vitro* and *in vivo* experiments have clarified Fer-1’s protective effect on the DR retina and its ferroptosis-regulating mechanism, corroborating the clinical potential of ferroptosis-targeted intervention, critical limitations remain in its targeting specificity, potential off-target risks and clinical translational potential of preclinical models. Insufficient *in vivo* stability of Fer-1 directly restricts its clinical translatability, pending further validation and optimization. Though defined as a specific ferroptosis inhibitor, Fer-1 is a blanket lipophilic antioxidant; it hits all retinal cells alike, lacks aimed action on DR-critical cells, and can blur normal redox signaling. Intravitreally, it may accumulate and turn toxic at high doses. Systemically, it risks upsetting whole-body iron homeostasis, adding off-target liability. Furthermore, the lack of large-sample long-term experimental data results in undefined efficacy and safety of Fer-1 for DR at different stages, further reducing the practical translational value of this agent.

### 5.2 Mechanism of iron chelators in improving DR by regulating iron homeostasis

Iron chelators are the main drugs for treating methemoglobinemia. They can chelate excess iron ions to reduce iron-catalyzed free radical reactions, thereby alleviating oxidative stress damage to retinal cells. Deferoxamine (DFO) can directly chelate iron elements and has been approved by the US Food and Drug Administration for clinical treatment to reduce the bioavailability of iron (234). An experimental study showed that in the mature genetic model of type 2 diabetes, the db/db mouse, after systemic administration of the iron chelator deferoxamine by intraperitoneal injection for 12 weeks, significant reductions in inflammatory markers such as IL-1β and TNF-α were observed, and retinal electrophysiological function was improved. At the same time, it can also increase the levels of antioxidants such as GPX4, GSH, and SOD, and significantly reverse pathological changes caused by iron homeostasis disorders (136). Collectively, these studies firmly confirm that iron chelators represented by DFO alleviate DR pathogenesis by regulating iron homeostasis and inhibiting oxidative stress and inflammation, and the FDA-approved clinical status of DFO further solidifies the translational feasibility of iron chelation therapy. However, these findings are mostly limited to single models, lacking data on distinct DR stages, ocular targeted delivery, long-term safety and efficacy, and comparisons with first-line clinical therapies. Coupled with the absence of clinical outcome measures and human clinical trial evidence, their direct clinical translation is restricted, highlighting an urgent need for more clinically relevant studies to optimize intervention strategies and clarify the actual therapeutic value of iron chelators in DR patients.

### 5.3 Research progress of natural products targeting ferroptosis for DR treatment

In recent years, natural compounds, with their structural diversity, chemical diversity, and rich biological activities, have become an important source for drug development. These breakthroughs have promoted the application research of natural products in the treatment of diabetic retinopathy (235). In recent years, it has been confirmed that natural products such as resveratrol, berberine, isoquercetin, platycodin D, and astragaloside IV can alleviate DR by inhibiting ferroptosis. AS-IV has been proven to effectively inhibit the death of RPE cells in DR induced by streptozotocin. Cellular changes show that AS-IV can inhibit cell damage caused by a high-sugar environment by increasing the expression of miR-128 (186). Studies have found that resveratrol can inhibit the apoptosis of retinal ganglion cells by inhibiting the Nrf2/HO-1 pathway and reducing retinal oxidative stress, thereby achieving the goal of treating DR (235). In addition, it has been found that most natural polyphenols play an antioxidant role by activating the Nrf2 and GPX4-related pathways, thereby playing an important role in the treatment of diabetes and its complications (228, 229, 236). Platycodin D (PLD) is a triterpenoid saponin isolated from the dried roots of Asarum heterotropoides. Studies have shown that PLD can exert therapeutic effects on DR *in vivo* by downregulating the ferroptosis-related TLR4/MyD88/NF-κB p65 pathway and upregulating the Nrf2/HO-1 pathway (226). In the high glucose cultured HRMECs model, intervention with berberine can upregulate the expression of Nrf2, HO-1, GPX4, and FSP1 while inhibiting ACSL4 (221). Isoquercetin can significantly alleviate retinal damage in mice with DR, and *in vitro* studies have confirmed that its protective effect is mediated by inhibiting the p53 signaling pathway (227). Pharmacological studies have also found that the total flavonoids (TFA) extracted from the flowers of Portulaca oleracea have functions similar to Fer-1. This component exerts its effects by activating the Nrf2 signaling pathway, reducing iron deposition, and enhancing antioxidant capacity (237). 1,8-Cineole reduces ferroptosis in retinal epithelial cells by inhibiting the PPAR-γ/TXNIP pathway (225). However, subsequent studies should focus on extract purification and structural modification to improve bioavailability, clarify precise molecular mechanisms with clinical samples, and conduct standardized preclinical studies with clinical outcomes as endpoints, so as to fully validate their therapeutic value and accelerate clinical translation for DR treatment.

## 6 Conclusions and perspectives

The pathophysiological mechanisms of DR are highly complex. Ferroptosis, a novel form of programmed cell death, involves intricate molecular mechanisms. This process is driven by iron-dependent peroxidation of phospholipids and is regulated by various cellular metabolic activities and signaling pathways. Although the pathophysiological role of ferroptosis is still being explored, its close association with DR has been confirmed. In relevant experiments, some ferroptosis inhibitors and iron chelators have shown good regulatory effects, and inhibiting ferroptosis can effectively prevent and delay the progression of the disease. Numerous natural products and drugs against ferroptosis also provide new ideas and targets for the treatment of DR. However, current research still has numerous critical shortcomings and bottlenecks to be addressed, which urgently require objective scrutiny and in-depth refinement. Most studies focus on the global regulation of blood-retinal barrier (BRB) function, and the cell-specific regulatory characteristics of ferroptosis in distinct retinal cell subtypes remain undefined, making it difficult to accurately distinguish the differential roles of cell-specific ferroptosis across various DR stages. The understanding of the specific regulatory mechanisms of organelles such as mitochondria and endoplasmic reticulum in ferroptosis, as well as the inter-organelle crosstalk network, is still limited. Additionally, the interactive crosstalk mechanisms between ferroptosis and other regulated cell death pathways have not been fully elucidated, failing to clarify the core position and synergistic mode of ferroptosis in the complex retinal cell death network of DR. In summary, more systematic and in-depth mechanistic and translational research is urgently needed to define the pivotal role of ferroptosis in DR pathogenesis and therapy. Comprehensive and in-depth dissection of the regulatory mechanisms, signaling networks and pathophysiological significance of ferroptosis can not only more clearly reveal its intrinsic association with DR, but also hold great promise for identifying novel diagnostic and therapeutic targets for DR, further deepen the understanding of the intricate pathophysiological processes of DR, and lay a fundamental theoretical foundation for developing precise and efficient ferroptosis-targeted therapeutic regimens for DR.
